# Prediction models for stroke-associated sarcopenia: a scoping review

**DOI:** 10.3389/fneur.2026.1934093

**Published:** 2026-10-30

**Authors:** Ying Xia, Ling Huang, Xin Li, Xiaolu Zhong

**Affiliations:** 1 Three Gorges University College of Medicine and Health Sciences, Yichang, Hubei, China; 2 Renhe Hospital Affiliated to China Three Gorges University, Yichang, Hubei, China

**Keywords:** prediction model, predictors, sarcopenia, scoping review, stroke

## Abstract

**Objective:**

To systematically map the study designs, model development methods, predictive performance, and predictors of risk prediction models for sarcopenia in stroke patients, and to identify current research status, gaps, and future directions in this field.

**Methods:**

This review followed the Arksey and O’Malley five-stage framework. Nine databases (PubMed, Web of Science, Cochrane Library, Embase, CINAHL, CNKI, Wanfang, VIP and SinoMed) were searched from inception to October 2025. Two reviewers independently screened and extracted data using a form based on the JBI Manual, with methodological classification guided by the Transparent Reporting of a multivariable prediction model for Individual Prognosis Or Diagnosis (TRIPOD) statement. A narrative synthesis approach was adopted.

**Results:**

A total of 12 studies (2022–2025) comprising 15 prediction models were included, with sample sizes ranging from 80 to 1,226 and stroke-associated sarcopenia (SAS) prevalence rates of 15.8–65.8%. Nine studies were conducted in China, and the most common study designs were case–control and cross-sectional studies. Most studies employed parametric methods, predominantly logistic regression, whereas two studies also employed non-parametric machine learning methods, including decision trees, random forest, and XGBoost. And only one study performed true external validation. Seven studies of multivariable diagnostic prediction models reported AUC values ranging from 0.743 to 0.970, and four studies evaluating the diagnostic accuracy of single or few biomarkers reported AUC values ranging from 0.681 to 0.830. Calibration metrics were inconsistently reported across studies. Predictors spanned six dimensions: demographic and lifestyle factors, muscle morphology and body composition, medical history and comorbidities, neurological and cognitive-psychological factors, nutritional and feeding-related factors, and laboratory indicators.

**Conclusion:**

Current SAS risk prediction models are predominantly single-center, small-sample studies that generally lack external validation and calibration reporting, limiting their clinical translational value. Although previous systematic reviews have addressed SAS prediction models, this review also summarized exploratory single-biomarker studies, including those evaluating inflammatory-immune markers such as CAR, NLR, and HMGB1. These markers may provide new directions for SAS risk prediction; however, each is supported by only one small study and should be considered promising predictors rather than validated predictors. Future research should conduct multicenter, prospective studies using standardized sarcopenia diagnostic criteria, adhere to the TRIPOD statement to construct multidimensional prediction models with external validation, and facilitate the translation of risk screening into clinical practice.

**Systematic review registration:**

https://doi.org/10.17605/OSF.IO/BWKMR.

## 1 Introduction

According to the Global Burden of Disease (GBD) Report, stroke has become the second leading cause of death worldwide and the third leading cause of long-term acquired disability among adults (1). The high incidence of post-stroke disability leaves many survivors with persistent neurological deficits, which not only significantly impairs their quality of life, but also places a substantial caregiving and economic burden on their families and society (2).

Sarcopenia is widely regarded as a progressive and generalized skeletal muscle disorder, characterized by the loss of muscle strength, muscle mass, and physical function (3). Based on etiology, sarcopenia can be broadly divided into primary and secondary types. Primary sarcopenia is mainly driven by age-related degenerative processes and predominantly affects individuals aged 65 years or older (4). By contrast, secondary sarcopenia generally develops in the presence of specific underlying or systemic conditions, and is frequently linked to diseases such as type 2 diabetes mellitus and cardiovascular disease (5, 6). In patients with stroke, neurological deficits and related complications, including dysphagia, may directly or indirectly induce structural, metabolic, and functional changes in skeletal muscle. If these alterations remain unresolved, they may evolve into progressive muscle wasting and tissue remodeling, consequently raising the risk of developing secondary sarcopenia (7).

Stroke-associated sarcopenia (SAS) is commonly defined as a clinical syndrome marked by reductions in skeletal muscle mass, muscle strength, and/or physical function occurring after stroke-related brain injury (8). Multiple studies have previously reported a high prevalence of sarcopenia in stroke patients, with estimates ranging from 32.1 to 68.4% (9–12). Stroke patients with comorbid sarcopenia often experience progressive muscle wasting and malnutrition due to restricted mobility and disease-related catabolism. This vicious cycle heightens the risk of infection, extends hospital stays, and ultimately worsens overall prognosis (13–15). Therefore, early identification of patients at high risk of developing sarcopenia following stroke, along with timely implementation of preventive and therapeutic interventions, is of paramount importance.

Risk prediction models translate traditional population-based data into individualized risk probabilities, providing clinicians and patients with quantified evidence for decision-making (16). Unlike traditional analyses of influencing factors, risk prediction models integrate multiple independent risk factors to estimate the probability of an outcome, thereby facilitating early clinical intervention and individualized treatment (17).

As a quantitative tool for risk assessment, risk prediction models have garnered increasing attention in the field of stroke-associated sarcopenia. Numerous studies have explored the influencing factors for sarcopenia in stroke patients, including age (10, 18–22), body mass index (19, 20, 22), smoking (22), nasogastric feeding (10, 18, 20, 21), NIHSS score (10, 20, 21), history of pneumonia (19, 20), albumin level (18–20), hemoglobin level (18, 19) and nutritional status (20). These findings have laid the foundation for developing risk prediction models for stroke-associated sarcopenia.

However, to date, uncertainty persists concerning the prediction models applicable to the stroke population at risk of sarcopenia, the predictor variables they include, and their overall performance. Accordingly, we conducted this scoping review.

## 2 Materials and methods

This review followed the five-step framework developed by Arksey and O’Malley (23). The five steps are: (1) clarifying the research question and defining the scope; (2) searching for relevant literature; (3) screening studies for eligibility; (4) extracting and synthesizing data; (5) summarizing and reporting the findings.

### 2.1 Identifying the research question and scope

This scoping review was guided by the following three research questions:

What are the types (diagnostic vs. prognostic) and study populations of prediction models for sarcopenia in stroke patients?
What are the model development methods and predictive performance of prediction models for sarcopenia in stroke patients?
What predictive factors are included in prediction models for sarcopenia in stroke patients?

Inclusion criteria

Population: Adult patients (≥18 years) with stroke, diagnosed according to any available diagnostic criteria.

Concept: Development or validation of prediction models or algorithms, or assessment of individual predictors, for predicting the occurrence of sarcopenia in patients with stroke.

Context: No restrictions were applied regarding clinical setting, geographical location, or healthcare system.

Exclusion criteria

The following types of publications were excluded: animal studies, reviews, study protocols, and meta-analyses.

### 2.2 Identifying relevant studies

A systematic search was conducted across the following Chinese and English databases: China National Knowledge Infrastructure (CNKI), Wanfang Database, VIP Database for Chinese Technical Periodicals, SinoMed, PubMed, Web of Science, Embase, Cochrane Library, and CINAHL. The search covered all records from database inception to October 30, 2025.

Taking CNKI as an example, the search strategy for Chinese databases was as follows: (stroke OR apoplexy OR cerebrovascular accident OR cerebral infarction OR intracerebral hemorrhage OR cerebral ischemia OR cerebral embolism OR cerebrovascular disease) AND (sarcopenia OR muscle loss OR muscle wasting syndrome OR muscular atrophy OR skeletal muscle loss OR muscle decline OR muscle mass OR muscle strength) AND (risk prediction OR risk prediction model OR prediction model OR diagnostic model OR risk score OR risk assessment OR clinical prediction model OR risk factor OR nomogram).

For English databases, the search strategy for PubMed is presented as a representative example; the complete search strategies are provided in the Supplementary materials.

### 2.3 Study selection

Retrieved records were imported into EndNote 21 for deduplication. Two reviewers (XY and ZXL) independently screened the literature against the inclusion and exclusion criteria. Any disagreements were resolved through discussion with a third reviewer (LX).

### 2.4 Data extraction and synthesis

A data extraction form was developed with reference to the JBI Manual for Evidence Synthesis (using the Joanna Briggs Institute template) (24). Based on the full texts of all included studies, the following information was extracted: author(s), year, country, study design, sample size and number of events, and model-related information, including statistical methods, model performance, development strategies, validation approaches, and final predictors. The included prediction model studies were methodologically classified in accordance with the TRIPOD statement and clinical prediction model methodology (25, 26). Data were extracted independently by two reviewers (XY and HL) to ensure that all necessary information was captured comprehensively and accurately. In addition, the risk of bias and applicability of the included prediction model studies were assessed using the Prediction model Risk Of Bias ASsessment Tool (PROBAST) (27). Two reviewers (LX and ZXL) independently evaluated the four PROBAST domains (participants, predictors, outcome, and analysis) and the three applicability domains (participants, predictors, and outcome). Any discrepancies were resolved through discussion or by consulting a third reviewer (HL).

### 2.5 Collating, summarizing, and reporting the results

Due to the heterogeneity of the data, a meta-analysis was not feasible. Therefore, we adopted a narrative synthesis approach to summarize the extracted data, present our results narratively, and supplement findings with figures and tables.

## 3 Results

A total of 1,379 records were identified through electronic database searches, and 1,041 remained after duplicate removal. Following title and abstract screening, 943 studies were excluded, leaving 98 for full-text review. Among them, 6 reports were unavailable. Additional exclusions were made due to ineligible populations (*n* = 4), lack of relevance (*n* = 58), and absence of a prediction model (*n* = 18). In total, 12 studies (28–39) were included in the final analysis (Figure 1).

**Figure 1:**
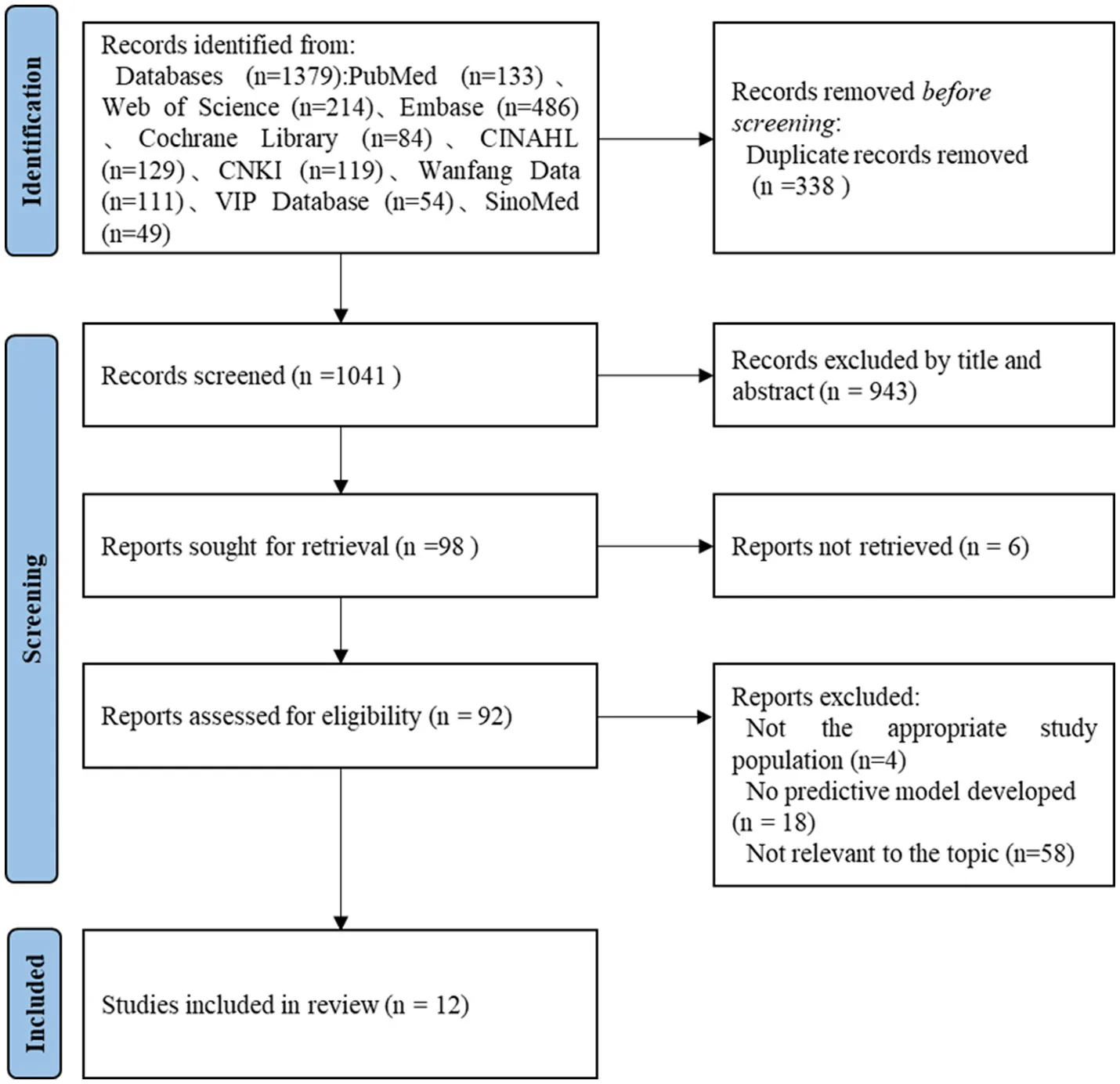
PRISMA flow diagram of study selection.

### 3.1 Types of models and study populations of prediction models for sarcopenia in stroke patients

The 12 included studies were published between 2022 and 2025. The majority of the studies were conducted in China (*n* = 9), followed by Turkey (*n* = 2) and Japan (*n* = 1). A total of 12 studies were included in this review, comprising five case–control studies, three cross-sectional studies, two cohort studies, and two prospective observational study. Eight studies (29, 31–35, 38, 39) developed multivariable diagnostic prediction models. The remaining four studies (28, 30, 36, 37) did not develop multivariable prediction models. This review included 12 studies comprising 15 models, with sample sizes ranging from 80 to 1,226 participants. The reported prevalence of stroke-associated sarcopenia ranged from 15.8 to 65.8% across studies using different diagnostic criteria (Table 1; Figure 2).

**Table 1:** Types and samples of models.

Author	Year	Country	Study design	sample size [training/validation (n)]	outcome events (%)	Outcome definition (Term → Post-stroke timing/assessment → Pre-stroke exclusion)	Assessment tool
Wang et al. (39)	2025	China	Prospective observational study	251/62	E:30.3 F:37.1	Term: “stroke-associated sarcopenia (SAS).” Timing/assessment: NR; clinically stable; first-ever stroke. Exclusion: excluded; pre-admission sarcopenia excluded by SARC-*F* > 4.	AWGS^a^
Yan et al. (38)	2025	China	Retrospective cohort study	794/159	E:37.5 F:38.4	Term: “stroke-related sarcopenia/secondary sarcopenia.” Timing/assessment: NR; inpatient/rehabilitation. Exclusion: excluded by recalled pre-stroke SARC-F ≥ 4.	AWGS 2019^b^
Se˘gmen et al. (37)	2025	Turkey	Retrospective cohort study	120	65.8	Term: “sarcopenia in cerebrovascular events (CVE)” defined by CSA-M. Timing/assessment: ICU admission; brain CT on admission. Exclusion: NS; prior CVE excluded.	CSA-M
Dogan et al. (36)	2025	Turkey	Prospective observational study	197	19.3	Term: “probable sarcopenia in acute ischemic stroke.” Timing/assessment: acute ischemic stroke within first 24 h of admission. Exclusion: NS.	SARC-F Low HGS
Kong et al. (35)	2024	China	Cross-sectional study	489	37.6	Term: “sarcopenia in senile patients with stroke.” Timing/assessment: NR; assessed during stable phase. Exclusion: NS.	SARC-F
Cheng et al. (34)	2024	China	Cross-sectional study	286	61.6	Term: “sarcopenia in patients with stroke.” Post-stroke timing/assessment: NR; at presentation (Oct–Dec 2023). Exclusion: NS.	SARC-F
Pu et al. (33)	2024	China	Case–control study	209	15.8	Term: “post-stroke sarcopenia.” Timing/assessment: NR; NHANES 2011–2018 cross-sectional. Exclusion: NS.	SMI
Chen et al. (32)	2023	China	Retrospective case–control study	80	25.0	Term: “secondary sarcopenia in elderly stroke patients.” Timing/assessment: NR; during hospitalization. Exclusion: NS.	AWGS 2019^b^
Zhu et al. (31)	2023	China	Case–control study	290	Not reported	Term: “muscle attenuation syndrome/sarcopenia in stroke patients” Timing/assessment: day 7 after admission when clinically stable; rehabilitation inpatients. Exclusion: NS.	2017 Chinese Expert Consensus on Sarcopenia^c^
Wu et al. (30)	2023	China	Retrospective case–control study	80	35.0	Term: “sarcopenia during convalescence of stroke.” Timing/assessment: convalescent phase; exact post-stroke timing NR. Exclusion: NS.	AWGS 2014^d^
Yao et al. (29)	2022	China	Case–control study	1,226	48.3	Term: “stroke-related sarcopenia.” Timing/assessment: first-ever stroke within 6 months; rehabilitation department; assessment within 24 h of admission. Exclusion: excluded by recalled pre-stroke SARC-F ≥ 4.	AWGS 2019^b^ SARC-F; Ishii; CC
Nagano et al. (28)	2022	Japan	Cross-n sectional study	465	49.5	Term: “sarcopenia after acute stroke.” Timing/assessment: acute stroke; rehabilitation admission at median 26 days post-onset; assessed within 3 days of admission. Eclusion: NS	SMI; BIA; Jamar; Yamada

AWGS = Asian Working Group for Sarcopenia diagnostic criteria; ^a^version and cutoffs not reported; grip strength, ASMI, and calf circumference used; ^b^ASMI <7.0 kg/m^2^ (men)/<5.4 kg/m^2^ (women); grip strength <28 kg (men)/<18 kg (women); gait speed <1.0 m/s, 5-chair-stand ≥12 s, or SPPB ≤9; low muscle mass plus strength or physical performance criterion; ^c^BIA ASMI <7.0 kg/m^2^ (men)/<5.7 kg/m^2^ (women); grip strength <26 kg (men)/<18 kg (women); 5-chair-stand >12 s; low ASMI plus grip strength and/or 5-chair-stand criterion. ^d^BIA ASMI <7.0 kg/m^2^ (men)/<5.7 kg/m^2^ (women), or DXA ASMI <7.0 kg/m^2^ (men)/<5.4 kg/m^2^ (women); grip strength <26 kg (men)/<18 kg (women); 6-m gait speed <0.8 m/s; low ASMI plus grip strength and/or gait speed criterion. SARC-F = Simple 5-item questionnaire (≥4 points); CSA-M = cross-sectional area of the masseter muscle (threshold <400 mm^2^ for men)/<300 mm^2^ for women; ROC cutoff (right <300 mm^2^, left <295 mm^2^); Low HGS: low handgrip strength (cut-offs: <27 kg for men, <16 kg for women); SMI = Skeletal Muscle Mass Index (cut-offs: <0.789 for men and <0.512 for women); CC = calf circumference; BIA = bioelectrical impedance analysis for muscle mass measurement; Jamar = dynamometer for grip strength measurement; Yamada = muscle mass calculated via the Yamada formula; E:development; F:validation; NR = not reported; NS = not specified.

**Figure 2:**
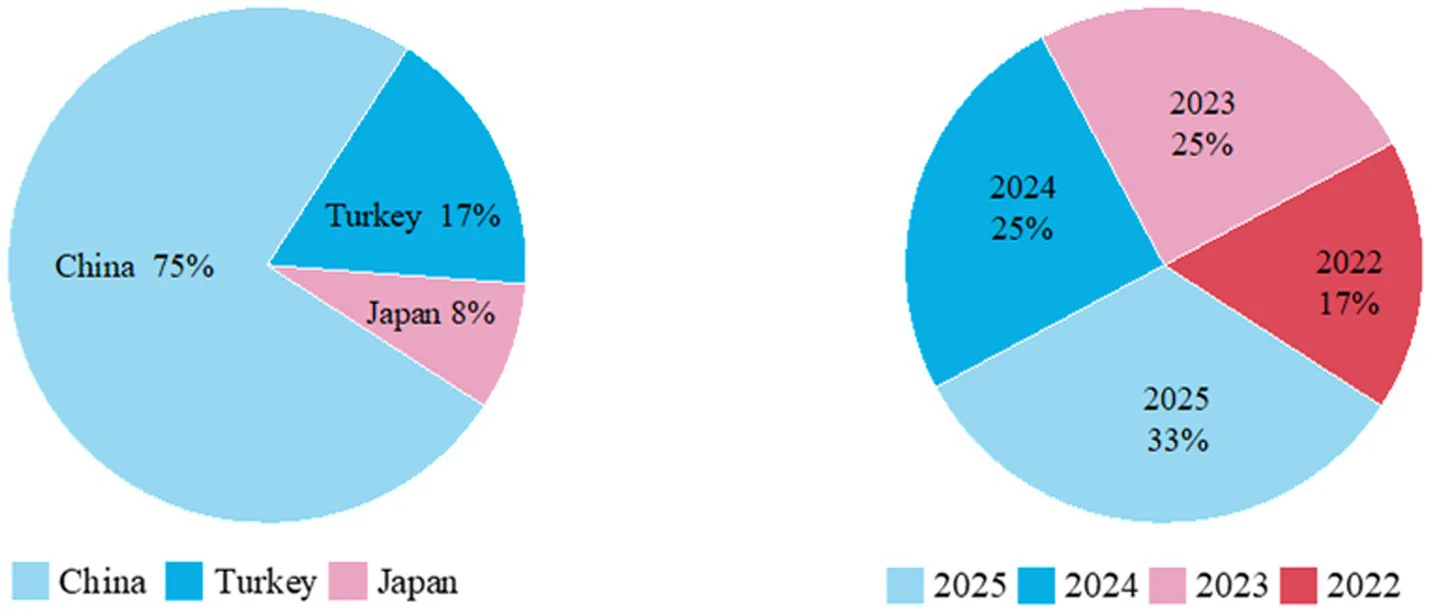
Publication year and country of the prediction models for sarcopenia in stroke patients.

### 3.2 Model development methods and performance of prediction models for sarcopenia in stroke patients

Among the included studies, the majority employed only parametric models, primarily logistic regression. One study (35) employed both logistic regression and a decision tree, and another study (38) compared logistic regression with random forest and XGBoost. With regard to model development and validation, seven studies (29, 31–35, 38) performed model development only, one study (39) performed both model development and geographical external validation, and four studies (28, 30, 36, 37) did not develop a multivariable prediction model. Model performance was primarily evaluated in terms of discrimination and calibration. Discrimination was assessed using the area under the curve (AUC) or the C-statistic, while calibration was evaluated using the Hosmer-Lemeshow test, calibration plots, calibration intercept, and calibration slope. Of the twelve included studies, eleven reported AUC values. Among them, seven studies (31–35, 38, 39) of multivariable diagnostic prediction models reported AUC values ranging from 0.743 to 0.970, and four studies (28, 30, 36, 37) reported AUC values ranging from 0.681 to 0.830. The remaining study (29) did not report AUC but reported a C-index of 0.761. Among the included studies, three studies (32, 34, 35) reported only the Hosmer-Lemeshow test, three studies (29, 31, 33) reported only calibration plots, two studies (38, 39) reported both the Hosmer-Lemeshow test and calibration plots and four studies (28, 30, 36, 37) did not report any calibration metrics. Six included studies performed DCA; Cheng et al. (34) reported beneficial threshold ranges of 0.15–0.95 in the training set and 0.34–0.98 in the testing set, Zhu et al. (31) and Yao et al. (29) reported a higher net benefit when the threshold probability was >6.4%, and the remaining three studies (33, 38, 39) only presented DCA curves showing that the model outperformed the “treat all” and “treat none” strategies. The remaining studies did not perform DCA (Table 2). In addition, we used PROBAST to assess eight prediction model studies; the remaining four were ineligible and excluded. Among the 8 included studies, only the study by Yan et al. (38) was rated as having an overall low risk of bias, whereas the remaining seven studies were rated as having an overall high risk of bias. All eight studies showed high overall applicability (Table 3).

**Table 2:** Model development methods and performance of models.

Study	Model development	AUC/C-statistic	Calibration	Model validation		TRIPOD analysis types
			Methods	Internal	External	
Wang et al. (39)	A	E: 0.891; F: 0.743	Hosmer-Lemeshow goodness-of-fit test; Calibration plot testing set	Bootstrap resampling	Geographical validation	Type 3
Yan et al. (38)	A, B, C	G: 0.805 (A) H: 0.816 (A) B: 0.796C: 0.780	Hosmer-Lemeshow goodness-of-fit test; Calibration plot testing set	Bootstrap resampling Temporal validation	Not reported	Type 2b
Segmen et al. (37)	A	CSA-M: Cut-off value for females<300 mm^2^:0.820; Males<295 mm^2^:0.830	Not assessed	Not reported	Not reported	Not applicable
Dogan et al. (36)	A	0.814	Not assessed	Not reported	Not reported	Not applicable
Kong et al. (35)	A, D	A:0.959; E:0.892 (D) M:0.826 (D)	Hosmer-Lemeshow goodness-of-fit test	Random split validation	Not reported	Type 2a
Cheng et al. (34)	A	0.835	Hosmer-Lemeshow goodness-of-fit test	Random split validation, Bootstrap resampling	Not reported	Type 2a Type 1b
Pu et al. (33)	A	E: 0.970; M: 0.900	Calibration plot testing set	Random split validation	Not reported	Type 2a
Chen et al. (32)	A	0.826	Hosmer-Lemeshow goodness-of-fit test	Not reported	Not reported	Type 1a
Zhu et al. (31)	A	0.933	Calibration plot testing set	Bootstrap resampling	Not reported	Type 1b
Wu et al. (30)	A	0.828	Not assessed	Not reported	Not reported	Not applicable
Yao et al. (29)	A	0.761	Calibration plot testing set	K-fold cross validation	Not reported	Not applicable
Nagano et al. (28)	A	Male: 0.726; Female: 0.681	Not assessed	Random split validation	Not reported	Not applicable

A: Logistic regression; B: Random forest; C: XGBoost algorithm; D: Decision tree; E: Training set; F: External validation set; G: Development cohort; M: internal. H: Internal temporal validation; TRIPOD analysis types: Type 1a, development only, with performance evaluated on the same data (apparent performance); Type 1b, development using the entire dataset, with internal validation using resampling; Type 2a, random split-sample development and validation; Type 2b, nonrandom split-sample development and validation; Type 3, development and validation using separate data; Type 4, validation of an existing (published) model using separate data.

**Table 3:** Summary of the risk of bias assessed by the PROBAST tool.

Author	Risk of bias				Applicability			Overall	
	Participants	Predictors	Outcome	Analysis	Participants	Predictors	Outcome	Risk of bias	Applicability
Wang et al. (39)	+	?	?	−	+	+	+	−	+
Yan et al. (38)	+	+	+	+	+	+	+	+	+
Kong et al. (35)	+	?	−	−	+	+	+	−	+
Cheng et al. (34)	−	?	−	−	+	+	+	−	+
Pu et al. (33)	−	−	−	−	+	+	+	−	+
Chen et al. (32)	−	+	−	+	+	+	+	−	+
Zhu et al. (31)	−	+	−	−	+	+	+	−	+
Yao et al. (29)	+	+	−	−	+	+	+	−	+

“+” indicates low risk of bias/high applicability; “−” indicates high risk of bias/low applicability; “?” indicates unclear risk of bias or applicability.

### 3.3 Predictors included in the prediction models

This review included 12 studies comprising 15 prediction models. Predictor frequencies were counted per study. Within the same study, Yan et al. (38) used the same predictors in their machine learning and logistic regression models, while the decision tree model of Kong et al. (35) did not include fall risk, unlike their logistic regression model. The frequency distribution of predictors is as follows:

Patient demographic and lifestyle factors: Five studies included age, four included BMI, three included smoking history, and one included exercise habits.

Muscle morphology and body composition: Masseter muscle cross-sectional area (CSA-M), temporal muscle thickness (TMT), waist circumference (WC) and right calf circumference (CC) were each included in one study.

Patient medical history and comorbidities: Three studies included history of diabetes, each of hyperlipidemia, the number of prior strokes and pneumonia was included in one study.

Neurological function and cognitive-psychological factors: Four studies included the NIHSS score, two included limb dysfunction, two included cognitive impairment, and one study each included dysphagia, sleep disorders, activities of daily living and anxiety.

Nutritional status and feeding-related factors: Three studies included tube feeding status, and three studies included nutritional status (The instruments and cut-off values used to assess nutritional status differed across studies; details are provided in the footnotes of Table 4).

**Table 4:** Risk predictors of the models.

Study	Number of predictors	Predictors
Wang et al. (39)	5	Body mass index (BMI), Stroke history, National Institutes of Health Stroke Scale (NIHSS) score, Serum albumin, Nasogastric tube placement
Yan et al. (38)	7	Age, Motor dysfunction, Diabetes mellitus, Nasogastric feeding, Body mass index (BMI), NIHSS score, C-reactive protein (CRP)
Segmen et al. (37)	1	Masseter muscle cross-sectional area (CSA-M)
Dogan et al. (36)	3	C-reactive protein-to-albumin ratio (CAR), Age, NIHSS
Kong et al. (35)	6	Age, Smoking, Activities of daily living (ADL), Nutritional status^a^, Fall risk, Exercise habits
Cheng et al. (34)	2	NIHSS score, C-reactive protein (CRP)
Pu et al. (33)	8	Sex, Body mass index (BMI), Height, Alkaline phosphatase (ALP), Total calcium, Creatine phosphokinase (CPK), Hemoglobin, Waist circumference
Chen et al. (32)	6	Age, Body mass index (BMI), Nutritional status^b^, Bone mineral density, Hyperlipidemia, Number of strokes
Zhu et al. (31)	6	Age, Smoking, Diabetes mellitus, Nutritional status^c^, Sleep disorder, Cognitive function
Wu et al. (30)	3	High mobility group box 1 (HMGB1), Albumin (Alb), Neutrophil-to-lymphocyte ratio (NLR)
Yao et al. (29)	13	Reduced muscle strength, Smoking, Lesion located in the temporoparietal lobe, Diabetes mellitus, Aphasia, Anxiety, Cognitive impairment, Dysphagia, Duration of nasogastric tube placement, History of coma, Pneumonia, Right calf circumference, Duration of rehabilitation treatment
Nagano et al. (28)	1	Temporal muscle thickness (TMT)

^a^Assessed using the Mini Nutritional Assessment-Short Form (MNA-SF); scores 0–11 indicated abnormal nutritional status and 12–14 indicated normal status. ^b^Assessed using the Modified Quantitative Subjective Global Assessment (MQSGA) score; the specific cut-off was not reported, and the score was analyzed as a continuous variable. ^c^Assessed using the Subjective Global Assessment (SGA); grade A, well nourished; grade B, mild-to-moderate malnutrition; grade C, severe malnutrition.

Laboratory indicators: Two studies included C-reactive protein (CRP), two studies included albumin (Alb), and one study each included hemoglobin (HGB), C-reactive protein-to-albumin ratio (CAR), high mobility group box 1 (HMGB1), creatine kinase (CK), and neutrophil-to-lymphocyte ratio (NLR). Notably, CAR, NLR, and HMGB1 are described as promising predictors that require further validation. Details of the predictors are presented in Figure 3 and Table 4.

**Figure 3:**
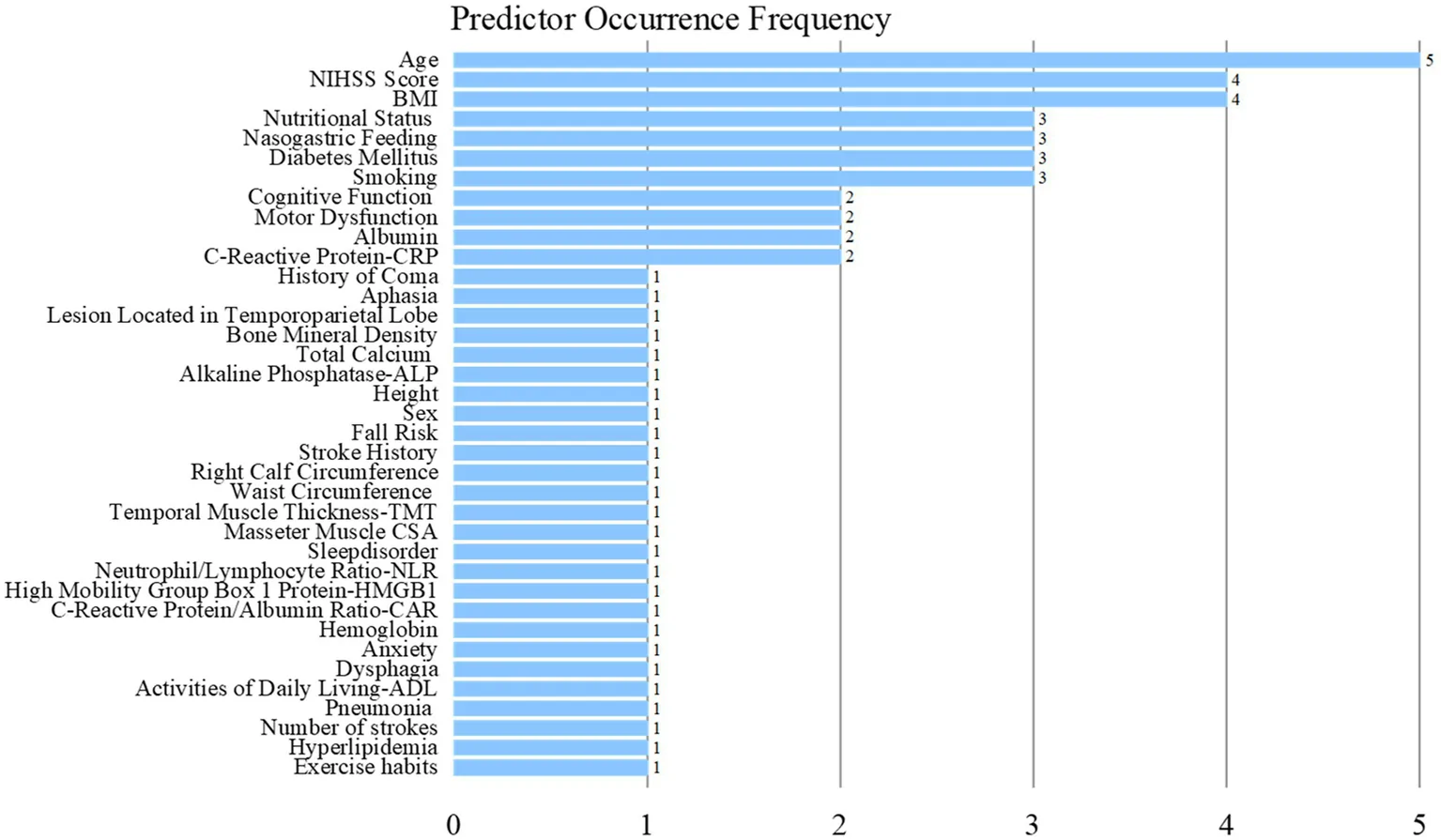
Frequency of predictors in the included models.

## 4 Discussion

This scoping review mapped the current landscape of risk prediction models for sarcopenia in stroke patients, covering study design types, model development methods, model performance, and predictors. Although there is considerable research interest in risk prediction models, studies focusing on risk prediction for sarcopenia among stroke patients remain limited; only 12 studies were included in this review. Notably, all studies were published within the last five years, indicating that risk prediction models for sarcopenia in stroke patients are receiving growing attention and recognition.

Due to substantial heterogeneity across the included studies, it was challenging to draw unified conclusions. Accordingly, this scoping review identified several key themes instead.

Globally, the prevalence of SAS varies considerably (9, 10, 18, 40–43), warranting further discussion. Notably, East Asian countries and regions, including Japan, China, and Korea, have been the major contributors to SAS research (44). In this scoping review, the total sample sizes ranged from 80 to 1,226, and the reported prevalence of SAS ranged from 15.8 to 65.8%, with substantial heterogeneity across studies. This heterogeneity may be attributable to variations in sarcopenia assessment methods, differences in countries or regions where studies were conducted, and disparities in the age ranges of study populations. Among these, variations in assessment methods are particularly important because they can influence prevalence estimates. For example, SARC-F is a screening questionnaire rather than a diagnostic criterion; therefore, studies using SARC-F identify individuals with probable or at-risk sarcopenia rather than confirmed sarcopenia. Consequently, prevalence estimates based on SARC-F are not equivalent to those based on AWGS or other diagnostic criteria. The reported prevalence range may therefore reflect differences in outcome definitions rather than true differences in disease burden. Most studies included in this review were conducted in China, which may be attributed to China’s increasingly severe population aging challenge. As a condition closely associated with advancing age, the early identification and prevention of sarcopenia carry significant public health implications (45–47). Therefore, Chinese researchers have prioritized developing valid prediction models and identifying relevant risk factors, aiming to provide an evidence-based early intervention against sarcopenia. This practical demand may partially explain the disproportionately high concentration of related studies conducted in China.

The risk prediction models for sarcopenia in stroke patients included in this scoping review predominantly employed traditional logistic regression, while a few studies (35, 38) also explored machine learning methods, such as random forest, decision trees and XGBoost. Parametric models assume a predefined relationship between predictors and the outcome (typically linearity on the log-odds scale) and rely on assumptions such as independence of observations. These assumptions make estimation more straightforward and enhance interpretability. Specifically, regression coefficients can be converted into odds ratios, and the model can be translated into a nomogram or risk score. Non-parametric machine learning methods do not assume a fixed functional form, so they can capture complex non-linear relationships and interactions. However, while non-parametric models generally fit the training data more closely, they do not necessarily generalize better and are more susceptible to overfitting in small samples. Therefore, machine learning models, in particular, require rigorous internal and external validation before clinical application. Furthermore, the strategy of univariate screening followed by multivariate modeling has inherent limitations: it may omit variables that are meaningful in multifactorial interactions, and variable selection results tend to be unstable in small- to moderate-sized samples. Future research could adopt a clinically informed variable selection framework rather than relying solely on statistical significance.

Regarding model performance, among the eight studies that developed multivariable diagnostic prediction models, these models demonstrated favorable predictive ability (AUC > 0.7). These findings indicate that these models generally have good diagnostic discrimination for prevalent sarcopenia in patients with stroke. However, according to the PROBAST assessment, only the study by Yan et al. had an overall low risk of bias, whereas the remaining seven studies had a high risk of bias. The outcome and analysis domains accounted for most of the bias concerns. Within the outcome domain, inconsistent definitions of sarcopenia and missing information on blinding were the primary sources of high risk. In the analysis domain, high risk stemmed mainly from small samples, too few outcome events, unreported handling of missing data, inadequate validation, and overfitting. High or unclear risk also emerged in the participants and predictors domains for some studies, largely due to study design and unblinded predictor assessment. Therefore, although these models generally showed good diagnostic discrimination for prevalent sarcopenia in patients with stroke, they should be interpreted with caution and require further external validation before routine clinical use. In contrast to the model-based studies, the four non-model studies evaluated only single or few biomarkers (AUC 0.681–0.830) and should be considered preliminary evidence for promising diagnostic biomarkers rather than validated model performance. Taken together, this review identified several common limitations. First, the majority of models were derived from single-center retrospective or cross-sectional studies, and only Wang et al. conducted external validation. Second, the sample sizes were generally small (mostly < 300 cases); several studies had EPV < 20 (Chen et al., Pu et al., and Wang et al.), raising concerns about overfitting, which may inflate apparent performance and reduce reproducibility. For instance, Wang et al. observed a decrease in AUC from 0.891 to 0.743 during external validation, likely reflecting overfitting. By comparison, Kong et al., Yan et al., and Yao et al. had larger samples and more events (EPV ≥ 20), which may have reduced the risk of overfitting. Third, although all models reported discrimination (AUC/C-statistic), calibration metrics were inconsistently reported: only a few studies reported both calibration plots and the Hosmer-Lemeshow test, some reported only the Hosmer-Lemeshow test, and others reported no calibration metrics. DCA was also underreported, thereby limiting the clinical translational value of these models. Finally, the included studies exhibited heterogeneity in sarcopenia diagnostic criteria, which may have compromised the consistency of predictor identification. Because different SAS definitions represent different target conditions and produce different case mixes, misclassification, and event rates, AUC, calibration, and clinical utility are not directly comparable across definitions. Model performance should therefore be compared cautiously, ideally only within studies using the same reference standard. This heterogeneity limits the comparability of prevalence estimates and model performance across the included studies. Future studies should use standardized diagnostic criteria (e.g., AWGS) and adhere strictly to the Transparent Reporting of a Multivariable Prediction Model for Individual Prognosis or Diagnosis (TRIPOD) statement to standardize the reporting of prediction models.

This scoping review included 12 original studies and systematically mapped the predictors of SAS occurrence. Compared with an existing systematic review (48), the present review incorporated a greater number of studies and covered a broader range of predictors. Previous studies have primarily focused on traditional predictors, including demographic factors, nutritional status, stroke-specific clinical indicators, and functional assessment tools among others. In contrast, the present review also summarized exploratory single-biomarker studies evaluating biomarkers such as the C-reactive protein-to-albumin ratio (CAR), high mobility group box 1 (HMGB1), and neutrophil-to-lymphocyte ratio (NLR); these studies may help broaden the dimensions of SAS risk prediction. However, each of these markers was supported by only one small study and should be considered promising predictors rather than validated predictors.

CAR, which integrates systemic inflammation and nutritional status, is a recently emerging composite biomarker (36, 49). CRP, an acute-phase inflammatory protein, is markedly elevated during the acute stage of stroke and mediates skeletal muscle atrophy by promoting protein catabolism and suppressing muscle synthesis signaling, such as the PI3K/Akt/mTOR pathway (50–52). Albumin levels, on the other hand, reflect the body’s nutritional reserves and protein synthesis capacity; hypoalbuminemia is closely associated with reduced muscle mass (53, 54). Evidence suggests (36) that CAR demonstrates favorable predictive performance for probable sarcopenia in patients with acute ischemic stroke (AUC = 0.814), holding promise as a convenient and efficient bedside screening tool for identifying patients at high risk of SAS.

NLR is a comprehensive indicator reflecting systemic inflammatory status and immune function. Following stroke, neuroimmune activation triggers extensive neutrophil recruitment, accompanied by a decline in T lymphocyte counts; an elevated NLR thus signifies a disruption in the pro-inflammatory/anti-inflammatory balance (55). Sustained hyperinflammation promotes muscle fiber protein degradation and ultimately leads to muscle loss by activating the NF-κB signaling pathway and upregulating muscle atrophy-related E3 ubiquitin ligases (MAFbx/Atrogin-1 and MuRF1) (51, 56). Studies have shown that NLR is an independent predictor of sarcopenia, and its cutoff value and clinical feasibility give it substantial potential for clinical application (57). HMGB1, a late-acting damage-associated molecular pattern (DAMP), is abundantly released into the circulation by necrotic cells and activated immune cells during post-stroke neuroinflammation.

HMGB1 binds to RAGE and TLR4, activating downstream inflammatory cascades that impair skeletal muscle satellite cell function and trigger muscle atrophy signaling pathways (58–60). Compared with NLR, HMGB1 exhibits greater mechanistic specificity, primarily reflecting the “remote effects” of neuroinflammation on skeletal muscle. This mechanism has not been thoroughly elaborated in previous systematic reviews, suggesting that HMGB1 may represent an important entry point for mechanistic research on SAS.

In addition, Seğmen (37) et al. included masseter muscle cross-sectional area (CSA-M) as a reliable marker and predictor of clinical prognosis, Nagano (28) et al. included temporal muscle thickness (TMT), and Yao (29) et al. included right calf circumference (CC)—all these studies have not been reported in other studies.

This review has several limitations. First, as a scoping review, it did not conduct quality appraisal or pool effect sizes across the included studies; consequently, the predictive performance and strength of evidence for each predictor require further validation. Second, the included studies used inconsistent diagnostic criteria for sarcopenia, which may have contributed to substantial heterogeneity among the developed prediction models. In addition, nutritional status was assessed using different instruments (e.g., MNA-SF, SGA, and MQSGA) with varying cut-offs, which may have led to inconsistent definitions, limited model comparability, and partly explained differences in predictive performance. Therefore, caution is warranted when selecting a model for a specific target population or clinical outcome. Third, we reclassified Segmen et al., Nagano et al., Wu et al., and Dogan et al. as studies that evaluated the diagnostic accuracy of one or a few promising biomarkers without developing or validating a multivariable diagnostic prediction model. These studies were presented separately from the multivariable prediction models. In the narrative synthesis, however, we still summarized findings from both groups together. This may have blurred the line between validated model performance and preliminary biomarker evidence, and it is a limitation of this review. In addition, in the study by Segmen et al., CSA-M served both as part of the outcome definition and as the sole predictor. This overlap probably inflated the reported AUC, so the predictive value of CSA-M needs careful interpretation. Future studies should examine these candidate biomarkers in multivariable prediction models, provided that the outcome definition is independent of the biomarker itself. Fourth, most of the included studies were conducted in Asia, particularly China; therefore, caution should be exercised when generalizing these findings to other populations. Finally, although we searched both published literature and grey literature, including theses, publication bias may still exist.

## 5 Conclusion

This scoping review systematically mapped the existing risk factors for SAS. Unlike previous reviews, this review incorporated emerging inflammatory-immune markers such as CAR, NLR, and HMGB1 into the systematic analysis, thereby broadening the dimensions of SAS risk prediction. However, current evidence for these markers remains insufficient, and most existing prediction models are derived from single-center, small-sample studies which generally lack external validation. Future research should conduct multicenter prospective studies using standardized diagnostic criteria; integrate inflammatory-immune, musculoskeletal metabolic, and simple morphological indicators to construct multidimensional combined prediction models with external validation; and explore the longitudinal associations between inflammatory-immune markers and SAS to elucidate the dynamic patterns. This will ultimately facilitate the translation of SAS risk screening into routine clinical practice.

## Data Availability

The original contributions presented in the study are included in the article/Supplementary material, further inquiries can be directed to the corresponding authors.
