# Supplementary material for: Prediction models for stroke-associated sarcopenia: a scoping review
Source: Front Neurol. 2026 Oct 30;17:1934093. doi: 10.3389/fneur.2026.1934093 (PMC13759348; doi:10.3389/fneur.2026.1934093)
Supplement: Supplementary file 1 [file Table_1.docx]

PubMed search strategies
